# Positive episodic future thinking and its impact on the perceived performance anxiety in performing artists

**DOI:** 10.3389/fpsyg.2025.1531435

**Published:** 2025-07-01

**Authors:** Benedikt Gers, Klara Estela Weber, Mareike Altgassen

**Affiliations:** Department of Development Psychology, Johannes Gutenberg University, Mainz, Germany

**Keywords:** episodic future thinking, performance anxiety, anxiety disorders, performing artists, nervousness, imagination

## Abstract

Performance anxiety is characterized by specific fears of social situations involving potentially being judged or scrutinized by others. Research on coping strategies has focused on breathing techniques, biofeedback training and the use of beta-blockers; less is known about the impact of positive episodic future thinking (i.e., the imagination of successful events in one’s personal future). Given previous evidence of beneficial effects of episodic future thinking on emotional well-being, we hypothesized that positive episodic future thinking may lead to a decrease of performance anxiety in performing artists. Fifty-four performing artists (27 higher and 27 lower performance anxious) filled in a Performance Anxiety Questionnaire and imagined three different performance related events (two envisioned situations focussed on the moments shortly before having to perform, interjected by the imagination of currently successfully performing on stage). Overall, the ‘higher performance anxious’ group showed higher perceived nervousness in all three envisioned events than the ‘lower performance anxious’ group [*F*(1,52) = 13.04, *p* < 0.001, *η*^2^ = 0.20]. Both groups showed a significant decrease in perceived nervousness during (*p* < 0.001) and after (*p* < 0.001) engaging in positive episodic future thinking, suggesting the intervention has similar anxiety-reducing effects on severely and less severely affected performing artists. Implications regarding positive episodic future thinking as a possible treatment for anxiety are discussed.

## Introduction

Performance anxiety is classified by the Diagnostic and Statistical Manual of Mental Disorders as a subcategory of social anxiety disorders (SAD) and refers to a specific fear or anxiety concerning social situations centered around receiving criticism or scrutiny from others (Association AP, 2013). Performance anxiety may manifest itself in different settings, ranging from sports performances (Niering et al., 2023), public speaking (Blöte et al., 2009) to the performing arts, such as acting (Liu, 2023) or music performance (Nicholson et al., 2015). Regarding performance anxiety in musicians, for instance as outlined by Steptoe cited by Kenny (2011), characteristics for bouts of performance anxiety are affective (e.g., feelings of anxiety and/or tension), cognitive (e.g., loss of concentration, misreading the music score), somatic (e.g., shortness of breath) and behavioral (e.g., tremors, failure of technique) symptoms, as well as concrete nervousness and stage fright (Zakaria et al., 2013). The cognitive and affective symptomatology is indicative of worrying, a thinking style representative of anxiety (Papageorgiou, 2006). Different strategies to cope with performance anxiety have been proposed over the years, ranging from anti-anxiety medication (such as benzodiazepines, serotonergic compounds, psychoactive beta-blockers, etc.) (Kuwabara et al., 2024), progressive muscular relaxation, breathing techniques, biofeedback and meditation, to hypnotic suggestion (considered to be a hypnotic relaxation component added to cognitive behavioral therapy) (Brooker, 2018) or cognitive therapy (Liu, 2023). Fishbein et al. (1988) reported that out of 2,212 professional orchestra musicians a total of 27% indicated to have used propranolol hydrochloride or another beta blocker variant to cope with their performance anxiety. Out of these 27, 70% had not been prescribed this medication, which should be of concern, as uncoordinated use or even abuse of beta-blockers can lead to the development of cardiovascular, respiratory and gastrointestinal symptoms, as well as depression, psychotic symptoms and memory loss (Patston and Loughlan, 2014).

Episodic future thinking refers to the ability to imagine and mentally simulate events that might take place in one’s personal future (Schacter et al., 2017); in a similar vein to hypnotic suggestion, episodic future thinking involves the generation of imaginative events on the basis of altering memories and perception (Kihlstrom, 1997). Thus, the person engaging in episodic future thinking essentially pre-experiences the future through imagination. Episodic Future Thinking has been to be influential to decision-making, such as by countering temporal discounting and allowing for a more positive re-appraisal of the far-sighted choice, as well as planning by boosting prospective memory (Schacter et al., 2017); additionally, application of episodic future thinking may enhance empathic abilities and prosocial attitudes. Importantly, episodic future thinking needs to be differentiated from worry or other types of non-directed imagery (Wu et al., 2015). Worrying constitutes a future-oriented thinking style involving negative contents; the perception of the situation is less detailed and more abstract. Worry, a key component of performance anxiety, is characterized by cognitive inflexibility (i.e., persevering with maladaptive patterns in the face of failure rather than coming up with alternative coping strategies), and anticipating potential threats as a result of an imagined potentially arising situation rather than on the task itself (Wu et al., 2015; Nolen-Hoeksema et al., 2008). For example, a person having to give a presentation might worry more about the reaction of the audience rather than the actual presentation they are in control of and know in detail. In contrast, episodic future thinking comprises detailed and specific imagery (Wu et al., 2015) that creates a vivid representation of that future event.

Previous research has shown that engaging in episodic future thinking may lead to beneficial effects on emotional well-being (Jing et al., 2017), decision-making and problem-solving (see Schacter et al., 2012). Imagining constructive future behaviors in episodic detail regarding worrisome events is related to improved psychological well-being and decreases anxiety toward those events (Jing et al., 2016). In general, research has been conducted on the influence of imagery in maintaining and exacerbating anxiety disorders, such as in patients with social phobia (Hirsch and Holmes, 2007) and musicians with performance anxiety (Hackmann et al., 2000); furthermore, experimental studies have been able to show a decrease in music performance anxiety through the use of guided imagery (Kim, 2002). A distinction has to be made here between the guided imagery typically employed within the cognitive-behavioral framework, focusing on more general and impersonal muscle relaxation (Finch and Moscovitch, 2016) and positive episodic future thinking, which demands a personalized construction of a potential future event. Simulating personalized future events as a measure to reduce performance anxiety has so far not been studied.

Regarding performance anxiety, anxious individuals find it easier to generate negative outcomes for future events (MacLeod et al., 1997) and tend to believe that these negative outcomes are more likely to occur (MacLeod et al., 1997). Liang et al. (2021) investigated the effects of episodic future thinking in socially anxious individuals, finding that highly anxious participants imagined anxiety-provoking scenarios more negatively and with less detail than less anxious participants. The results of Jing et al. (2017) may point to how actively engaging in positive episodic future thinking and therefore raising the awareness of such thoughts could help to reduce the perceived performance anxiety before and during a performance. Therefore, further research into positive episodic future thinking might find it to be beneficial to individuals who experience more performance-related anxiety. However, to date, no study has investigated the potentially differential beneficial impact of positive episodic future thinking (e.g., in terms of relieving symptoms) in groups of varying anxiety level.

## Objectives

The primary aim of this study was to investigate whether actively engaging in positive episodic future thinking regarding an impeding performance can reduce performance anxiety-related nervousness. We further aimed to examine whether there are differences in the effectiveness of such a brief episodic future thinking intervention in higher performance anxious and lower performance anxious individuals.

The central hypothesis of this study constituted that individuals with high levels of performance anxiety would exhibit higher levels of nervousness across all positive episodic future thinking imaginations than participants with lower levels of performance anxiety (Zakaria et al., 2013). Secondly, we predicted nervousness to be lowest, on average, after the second imagination (i.e., imagining giving a successful performance), followed by the third and, lastly, the initial imagination (i.e., both requesting participants to imagine the moment just before going on stage to perform). Furthermore, we expected that nervousness reduction from the first to the last imagination would be significantly stronger in individuals displaying high levels of performance anxiety as compared to less anxious individuals (Wu et al., 2015; Hirsch and Holmes, 2007). Lastly, we intended to additionally investigate the influence of professional status and extent of performance experience on the expected nervousness reduction as the result of positive episodic future thinking.

## Materials and methods

### Participants

Fifty-four performing artists (33 women, 21 men, aged 19–75, *M* = 37.09, *SD* = 15.28) trained as actors/actresses, singers, or instrumentalist (12 singers, 18 actors, 24 instrumentalists) participated in the study; written informed consent was obtained and critical for inclusion. Participants were recruited via the local participant pool as well as word of mouth. Inclusion criteria included being a native speaker of either German or English (as the study was conducted in either of these languages); to take part, participants needed to be a performing artist, selecting the group they identify with (*Actor/Instrumentalist/Singer*) as well as the number of years of experience they had., and were otherwise excluded from the study. Further exclusion criteria included a history or presence of neurological diseases, psychiatric disorders, alcohol or drug abuse/addiction, regular consumption of psychotropic medication (particularly anti-anxiolytics).

### Design

**Figure 1:**
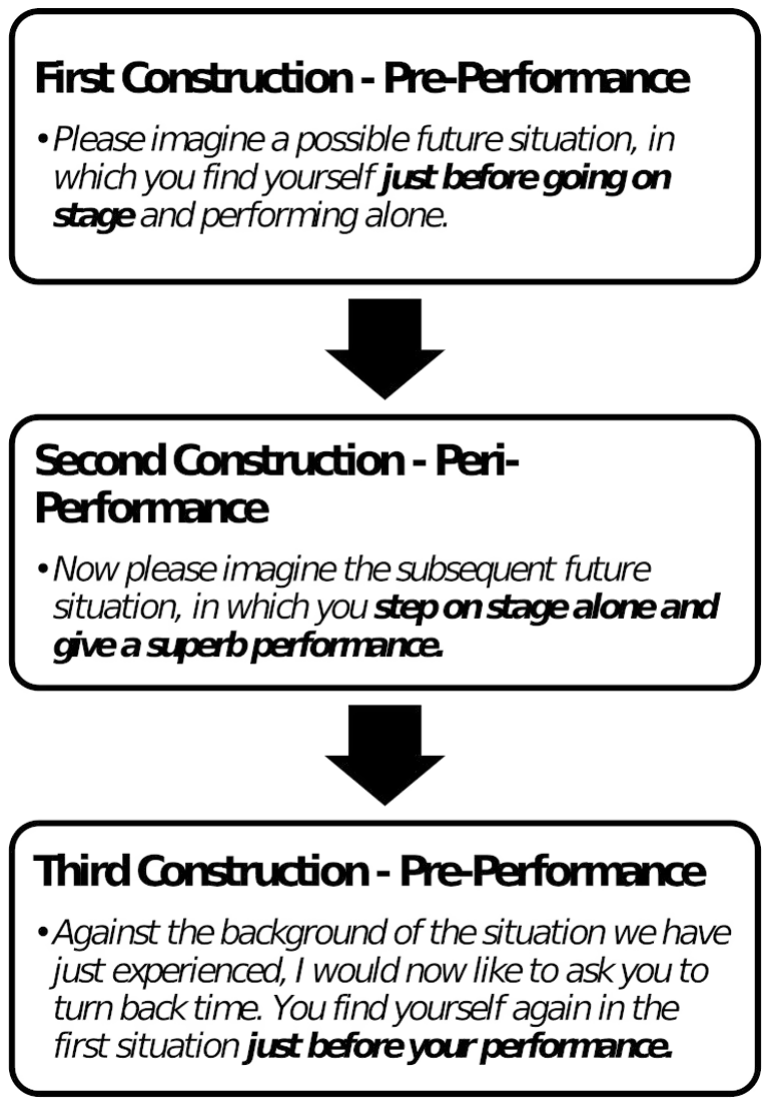
Illustration of experimental task order.

A 2 × 3 factorial design with the factors anxiety level (between subjects: high performance anxiety vs. low performance anxiety) by positive episodic future thinking event (within subjects: three episodic future thinking situations, two pre-performance imaginations interjected by one post-performance situation) was used in order to investigate the effects of anxiety level and positive episodic future thinking on perceived nervousness; the task design is displayed in Figure 1.

### Instruments

Demographic data was assessed via SoSci survey, an online questionnaire platform; this included information on age, gender, on-stage experience in years, professional or amateur status, as well as the number of performances per year. Furthermore, the online questionnaire included performance-related questions on a five-point Likert scale, ranging from 1 (far below average) to 5 (far above average), to assess qualifications and experience in terms of the participant’s respective performing art. The Performance Anxiety Questionnaire (PAQ) was employed in its original form in English (Cox and Kenardy, 1993), or the adapted German version “Der Bühnenangstfragebogen” (Fehm et al., 2002) (internal consistency Cronbach’s *α* = 0.88; retest-reliability 4–6 weeks: *r* = 0.86; validity: correlation with a measure of stage anxiety referring to a performance situation: *r* = 0.77, all *p*’s < 0.001) – depending on the native language of the participant (though it should be noted that measurement equivalence between the versions cannot be fully asserted). The PAQ consists of 20 statements, of which 10 describe cognitions and emotions during performance, while the remaining 10 describe somatic complaints (Fehm et al., 2002). The PAQ assesses how frequently the participant experiences each symptom, indicated on a five-point Likert scale, ranging from one (never) to five (always). In addition, two open-ended questions, asking the performers to explain how they cope with anxiety before and during a performance and whether they feel that their anxiety has a detrimental effect on their performance, were included, in order to allow for exploration of individual approaches to managing performance anxiety.

The experimental interview task was developed for this study, drawing on various tasks measuring Episodic Future Thinking. Self-reported assessments of the imagination along different criteria using Likert scales (Wu et al., 2015; MacLeod et al., 1997) and prompting the construction of the situation using sentences (Jing et al., 2017; Jing et al., 2016) have been employed within the Episodic Future Thinking paradigm. Ten questions to assess participants’ episodic future thinking ability as well as performance-related aspects were employed, and a five-point Likert scale, ranging from 1 (not at all) to 5 (very) was used. The criteria are outlined in Table 1.

**Table 1:** Description of self-assessment categories used in the Episodic Future Thinking task.

Category	Question
Imagination	On a scale from 1 to 5, 1 meaning “not at all” and 5 meaning “very well” – How well did you manage to imagine the situation?
Vividness	On a scale from 1 to 5, 1 meaning “vague” and 5 meaning “very vivid” – How vivid was the imagination of the situation?
Clarity	On a scale from 1 to 5, 1 meaning “not at all clear” and 5 meaning “very clear” – How clear was the place you imagined?
Coherence	On a scale from 1 to 5, 1 meaning “very fragmented” and 5 meaning “very clear” – How coherent did you perceive the scene to be?
Thoughts	On a scale from 1 to 5, 1 meaning “not at all” and 5 meaning “very well” – How well did you manage to imagine your thoughts in the situation?
Body signals	On a scale from 1 to 5, 1 meaning “not at all” and 5 meaning “very well” – How well did you manage to imagine your body signals in the situation?
Feelings	On a scale from 1 to 5, 1 meaning “not at all” and 5 meaning “very well” – How well did you manage to experience your feelings?
Nervousness	On a scale from 1 to 5, 1 meaning “not at all nervous” and 5 meaning “very nervous” – How nervous were you?
Valence	On a scale from 1 to 5, 1 meaning “very negatively” and 5 meaning “very positively” – How positively or negatively did you perceive your feelings?
Focus	On a scale from 1 to 5, 1 meaning “not at all focused” and 5 meaning “very focused” – How focused were you on your upcoming performance?

To assess performance-related nervousness, scores from the nervousness category in the Episodic Future Thinking task were used.

### Procedure

The recruitment period for this study ran from 22/06/2021 through 21/03/2023. After the participants submitted the online questionnaire, a date and time for the (digital) live interview was scheduled during which the brief Positive Episodic Future Thinking intervention was administered. Each interview lasted about 30 min and began with a practice imagination task unrelated to on-stage performance to familiarize the participant with the general form of the imagination. As part of this practice, the participant was asked to imagine in detail a situation at a swimming pool before a competition, which, while performance-related, was irrelevant to the type of on-stage performance we were interested in. The interview was divided into three blocks. In each of these blocks, the participants were asked to imagine engaging in a different, specific performance-related situation and to describe what they perceived. In the first situation the participants were asked to imagine a possible future situation right before stepping on stage and performing alone. In the second situation, the participants were asked to imagine stepping on stage alone and giving a superb performance. In the third situation the participants were asked to, once again, imagine a moment just before stepping on stage and performing alone. After describing each imagination, participants were asked to express in oral form their thoughts, feelings, and sensations by re-telling what they imagined, thereby ensuring that episodic future thinking was conducted, and to answer ten questions regarding the envisaged event; beyond the instruction outlined in Figure 1, this was unguided by the test administrator and no time limit was applied. After each imagination, participants rated themselves along the criteria outlined in Table 1. The interview ended with three final questions on how positively or negatively the participants rated the imagined stage experience; a five-point Likert scale ranging from 1 (very negatively) to 5 (very positively) was employed.

### Statistical analysis

In order to compare participants displaying higher levels of performance anxiety with those who show lower levels, the median score on the Performance Anxiety Questionnaire (PAQ) was used to perform a median split; this artificial categorization can be justified given the relevance of the PAQ within a clinical anxiety assessment, as well as the potential clinical implications toward future research (DeCoster et al., 2011). Post median split, participants were assigned to either the *Low Performance Anxiety* or *High Performance Anxiety* group. To assess the impact of the three imaginations on the reported levels of nervousness, and whether there is a group effect on the basis of reported levels of anxiety, a 2 × 3 mixed measures analysis of variance (ANOVA) was calculated, also allowing for the investigation of the progression of reported nervousness across all episodic future thinking tasks. Group differences along the lines of stage experience, performances per year and age were assessed using one-way ANOVAs. Furthermore, to examine to what extent professional status or performance experience predict a reduction in nervousness between the initial and final episodic future thinking construction, a linear mixed model was conducted using age, type of performer, stage experience, self-assessment of one’s artistic competence, and performances per year as fixed factors and individual differences as random effects.

## Results

The average total score reached in the Performance Anxiety Questionnaire (PAQ) was 54.09 (*SD* = 11.68). The median of 52 was used to split the sample in those with higher and those with lower performance anxiety (μ1 ≥ 52, μ2 < 52). One-way analyses of variance (ANOVA) were conducted to explore whether differences between high performance anxious and low performance anxious individuals could be found with regards to stage experience in years, performances per year and age. Means, standard deviation, sample sizes and factorial ANOVA results are shown in Table 2.

**Table 2:** Descriptive statistics split by performance anxiety and in total for stage experience, performances per year and age.

Dependent variable	Lower performance anxiety (*n* = 27)	Higher performance anxiety (*n* = 27)	Group	Significance	Effect size
	*M* (SD)	*M* (SD)	*F* (1,52)	*p*	ή^2^
Stage experience in years	19.07 (11.46)	18.41 (11.82)	0.044	0.834	0.001
Performances per year	22.63 (33.27)	22.52 (38.59)	0.000	0.991	0.000
Age	38.67 (16.10)	35.52 (14.49)	0.541	0.453	0.011

*M*, mean values; SD, standard deviation; *n* = sample sizes.

There were no significant differences between high and low performance anxious individuals in stage experience [*F*(1,52) = 0.44, *p* = 0.834], nor in performances per year [*F*(1,52) = 0.00, *p* = 0.991], nor in age [*F*(1,52) = 0.57, *p* = 0.453]. Means, standard deviation, sample sizes and factorial ANOVA results are shown in Table 3.

**Table 3:** Descriptive statistics for perceived nervousness across the imagined situations split along performance anxiety.

Dependent variable	Lower performance anxiety (*n* = 27)	Higher performance anxiety (*n* = 27)	Group	Significance	Effect size
	*M* (SD)	*M* (SD)	*F* (1,52)	*p*	ή^2^
1st construction	3.22 (1.05)	3.89 (0.89)	6.32	0.015	0.108
2nd construction	1.74 (0.76)	2.56 (1.22)	8.66	0.005	0.143
3rd construction	2.59 (1.08)	3.07 (1.10)	2.70	0.107	0.049

*M*, mean values; SD, standard deviation; *n* = sample sizes.

A 2 (high and low performance anxiety group) × 3 (the three future-centered imaginations) mixed measures analysis of variance (ANOVA) was used to investigate the impact of imagining specific performance-based situations on perceived nervousness across low and high performance anxious groups. The results revealed a significant main effect of performance anxiety level [*F*(1,52) = 13.04, *p* < 0.001, *η*^2^ = 0.20]; participants of the high performance anxiety group consistently reported higher levels of nervousness across all imaginations.

Furthermore, there was a significant main effect of positive episodic future thinking on perceived nervousness [*F*(2,106) = 29.79, *p* < 0.001, *η*^2^ = 0.36], with Bonferroni-adjusted post-hoc tests showing significantly higher nervousness scores in the first compared to the second (*p* < 0.001) and to the third (*p* < 0.001) constructed situation. Nervousness was also significantly higher in the third situation compared to the second (*p* = 0.001).

Assessing the interaction between performance anxiety level and the impact of the specific future-centered imagination, no significant effect was found [*F*(2,104) = 0.42, *p* = 0.662, *η*^2^ = 0.01]; indicating that any effects as a result of the imagination exercises were not significantly different between the anxiety groups. Using total scores across all three imaginations, group differences were further explored. The results are shown in Table 4.

**Table 4:** Descriptive statistics for assessed categories in the Episodic Future Thinking task split along performance anxiety.

Dependent Variable	Lower performance anxiety (*n* = 27)	Higher performance anxiety (*n* = 27)	Group	Significance	Effect size
	*M* (SD)	*M* (SD)	*F* (1,52)			*p*	ή^2^
Imagination	12.74 (1.85)	12.59 (1.58)	0.10	0.753	0.002
Vividness	11.48 (2.24)	12.07 (2.09)	1.01	0.320	0.019
Clarity	12.67 (2.09)	12.48 (2.38)	0.09	0.762	0.002
Coherence	10.85 (2.28)	11.04 (2.12)	0.10	0.759	0.002
Thoughts	12.11 (2.12)	12.04 (1,79)	0.02	0.890	0.000
Body signals	11.26 (2.35)	11.37 (2.12)	0.03	0.856	0.001
Feelings	11.56 (2.53)	12.30 (1.64)	1.63	0.207	0.030
Nervousness	7.56 (1.70)	9.52 (2.26)	13.04	<0.001	0.201
Valence	12.41 (1.67)	11.07 (1.69)	8.53	0.005	0.141
Focus	12.26 (1.70)	11.63 (2.34)	1.28	0.263	0.024

*M*, mean values; SD, standard deviation; *n*, sample sizes.

The high performance anxious group reported significantly higher levels of nervousness [*F*(1,52) = 13.04, *p* < 0.001, *η*^2^ = 0.20] and a more negative valence of thoughts [*F*(1,52) = 8.53, *p* = 0.005, *η*^2^ = 0.14]. There were no significant group differences in any of the other assessed categories.

Lastly, to explore potential influences of professionalism and experience on the effects of positive episodic future thinking, a linear mixed model analysis was conducted to further investigate potential influencing variable on the nervousness reduction between the first and third imagined situation; artist group and self-assessment as a professional were assessed as fixed effects, as well age, performances per year and previous stage experience. The model included random effects for participants. The results are shown in Table 5.

**Table 5:** Results for linear mixed model analysis using artist group, performances per year, stage experience, age and Professional Status to predict reduction in nervousness between the final and initial Episodic Future Thinking task condition.

Dependent Variable	Coefficient	S.E.	*t*	Sig.	95% Cl	
Constant	0.963	0.644	1.495	0.141	−0.333	2.259
Artist group = Actor	0.338	0.498	678	0.501	−0.664	1.340
Artist group = Instrumentalist	−0.334	0.487	−0.686	0.496	−1.312	0.645
Artist group = Singer	0^b^					
Professionalism = No	−0.027	0.507	−0.053	0.958	−1.047	0.993
Professionalism = Yes	0^b^					
Age	0.001	0.021	−0.350	0.728	−0.049	0.034
Stage experience	−0.007	0.026	0.005	0.996	−0.052	0.052
Performances per year	0.003	0.007	0.441	0.661	−0.010	0.016

^b^Parameter set to 0 due to redundancy.

The results show none of the examined fixed effects were significant (all *p* > 0.05). The model explained only 4.4% of the variance through fixed effects, which rose to 52.2% when including random effects.

## Discussion

The central objective of this study was to assess the potential benefits of positive episodic future thinking on reducing performance-related anxiety, and in particular the feeling of nervousness, in groups with either higher or lower levels of performance anxiety.

As expected (Cox and Kenardy, 1993), higher performance anxious individuals reported higher levels of nervousness in all three episodic future thinking events. Higher anxious individuals showed consistently higher levels of nervousness in the to be imagined episodic future thinking events of waiting on stage immediately ahead of having to perform as well as during imagining a superb performance on stage than less anxious individuals.

The general levels of nervousness across the three situations (Situations 1 and 3 referring to the moments imminently before performing, Situation 2 referring to giving a successful performance) followed the predicted curve: Nervousness across participants was highest in the first to be imagined event and lowest in the second; this implies (a) imagining having to perform soon induces anxiety, and (b) a detailed imagination of performing successfully significantly reduces those high levels of nervousness. Comparing the first and third imaginations, where participants imagined themselves shortly before performing on stage, participants displayed lower levels of nervousness in the third imagined event of being about to perform on-stage, which repeated the first imagination. This shows that engaging in positive episodic future thinking (during the second imagination) can decrease nervousness. This finding is in line with previous research which has shown that imagining constructive behaviors in episodic detail regarding worrisome events is related to improved psychological well-being and decreases anxiety toward those events; Jing et al. (2017) found that engaging in positive episodic future thinking toward the worrisome performance and experiencing the superb performance in detail through imagination led to a significant reduction in the perceived nervousness toward that event. Detailed episodic future thinking enhanced both specificity and tangibleness of an imagination pertaining to a future event, which then led to a reduction of the experienced anxiety toward that event (Schacter et al., 2012).

As indicated earlier, anxious individuals find it easier to generate negative outcomes for future events and believe negative outcomes to be more likely than positive ones (Kihlstrom, 1997; MacLeod et al., 1997). However, there is evidence that prompting anxious individuals to create positive alternative outcomes raises the probability ratings for those positive outcomes (Nolen-Hoeksema et al., 2008). Therefore, we expected in our third hypothesis that higher performance anxious individuals would experience a larger decrease in nervousness than lower performance anxious individuals after having been asked to imagine a positive future situation (i.e., a successful performance). In contrast to our prediction, however, no significant differences between the two groups were found in the present study. This suggests that positive episodic future thinking is equally effective for higher performance anxious as well as for lower performance anxious individuals. Further analysis into group differences only highlighted how the highly anxious participants reported more nervousness and more negative thoughts; as such, positive episodic future thinking appears to not have a stronger effect on perceived nervousness in highly performance anxious individuals.

In terms of other aspects potentially affecting the success of positive episodic future thinking in reducing nervousness or anxiety, a linear mixed model could not identify a significant fixed or random factor. This implies that neither amount of previous experience nor status as a professional artist affects to what extent positive episodic future thinking can reduce performance-related nervousness, implying that the level of perceived competence is not relevant. Similarly, there was no difference between artist category with regard to nervousness reduction; these findings suggest for positive episodic future thinking to be an effective countermeasure to pre-performance nervousness, and potentially performance-related anxiety, to a range of artists differing in performance type and experience.

Given the entirely subjective nature of self-rating measurements, leading to arguably less tangible results, future studies should combine self-rating scales with more objective psychophysiological measures, like the heart-rate variability when testing for nervousness and anxiety. Furthermore, this experiment was limited to positive episodic future thinking tasks before or during an *imagined* stage performance. In order to investigate whether episodic future thinking can reduce performance anxiety in actual performance situations, future studies should ask performers to engage in performance-related episodic future thinking shortly before an actual on-stage performance. A central limitation to this study is certainly any unknown measurement equivalence between the German and English versions of the PAQ. Additionally, in order to assess the relevance of constructing task-relevant situations, the inclusion of control groups imagining situations outside of the performance could be considered as a measure of comparison. In addition, future research could build upon this study by either designing intervention programs (i.e., treatment approaches manage performance anxiety) to be applied repeatedly to find potential lasting effects in stage performers, or assessing different target groups afflicted by strains of anxiety, both in clinical (e.g., persons with obsessive-compulsive personality disorder) and non-clinical (e.g., persons with pregnancy anxiety) samples. Lastly, future research may vote to enlist participants using random sampling to further strengthen the validity of the research.

In summary, this study showed that engaging in positive detailed episodic future thinking about an anxiously anticipated stage performance in the future has an impact on the perceived nervousness toward that event. Nervousness decreased during and after the positive episodic future thinking situation. Higher performance anxious individuals experienced higher levels of nervousness than lower performance anxious individuals in all conditions. Findings suggest that positive episodic future thinking may be beneficial in treating performance anxiety and reducing nervousness.

## Data Availability

The datasets presented in this study can be found in online repositories. The names of the repository/repositories and accession number(s) can be found at: https://www.psycharchives.org/en/item/5a8e39f0-d8be-41b0-9840-db88eba713fa.

## References

[ref1] Association AP (2013). Diagnostic and statistical manual of mental disorders, fifth edition. Arlington, VA: American Psychiatric Association.

[ref2] Blöte A. W. Kint M. J. Miers A. C. Westenberg P. M. (2009). The relation between public speaking anxiety and social anxiety: a review. J. Anxiety Disord. 23, 305–313. doi: 10.1016/j.janxdis.2008.11.007, PMID: 19117721

[ref3] Brooker E. (2018). Music performance anxiety: a clinical outcome study into the effects of cognitive hypnotherapy and eye movement desensitisation and reprocessing in advanced pianists. Psychol. Music 46, 107–124. doi: 10.1177/0305735617703473

[ref4] Cox W. J. Kenardy J. (1993). Performance anxiety, social phobia, and setting effects in instrumental music students. J. Anxiety Disord. 7, 49–60. doi: 10.1016/0887-6185(93)90020-L

[ref5] DeCoster J. Gallucci M. Iselin A.-M. R. (2011). Best practices for using median splits, artificial categorization, and their continuous alternatives. J. Exp. Psychopathol. 2, 197–209. doi: 10.5127/jep.008310

[ref6] Fehm L. Hille C. Becker E. (2002). Der Bühnenangstfragebogen (BAF) [German version of the Performance Anxiety Questionnaire]. Unpublished manuscript Technische Universität Dresden

[ref7] Finch K. Moscovitch D. A. (2016). Imagery-based interventions for music performance anxiety: an integrative review. Med. Probl. Perform. Art. 31, 222–231. doi: 10.21091/mppa.2016.4040, PMID: 27942702

[ref8] Fishbein M. Middlestadt S. E. Ottati V. Straus S. Ellis A. (1988). Medical problems among ICSOM musicians: overview of a national survey. Med. Probl. Perform. Art. 3, 1–8.

[ref9] Hackmann A. Clark D. M. McManus F. (2000). Recurrent images and early memories in social phobia. Behav. Res. Ther. 38, 601–610. doi: 10.1016/S0005-7967(99)00161-8, PMID: 10846808

[ref10] Hirsch C. R. Holmes E. A. (2007). Mental imagery in anxiety disorders. Psychiatry 6, 161–165. doi: 10.1016/j.mppsy.2007.01.005

[ref11] Jing H. G. Madore K. P. Schacter D. L. (2016). Worrying about the future: an episodic specificity induction impacts problem solving, reappraisal, and well-being. J. Exp. Psychol. Gen. 145, 402–418. doi: 10.1037/xge0000142, PMID: 26820166 PMC4792686

[ref12] Jing H. G. Madore K. P. Schacter D. L. (2017). Preparing for what might happen: an episodic specificity induction impacts the generation of alternative future events. Cognition 169, 118–128. doi: 10.1016/j.cognition.2017.08.010, PMID: 28886407 PMC5612915

[ref13] Kenny D. (2011). The psychology of music performance anxiety. New York: OUP Oxford.

[ref14] Kihlstrom J. F. (1997). Hypnosis, memory and amnesia. Philos. Trans. R. Soc. Lond. Ser. B Biol. Sci. 352:1727. doi: 10.1098/rstb.1997.0155, PMID: 9415925 PMC1692104

[ref15] Kim S. Y. (2002). The effect of guided imagery and preferred music listening versus guided imagery and silence on musical performance anxiety. Denton, Texas: Texas Woman's University.

[ref16] Kuwabara A. Olson E. M. Stanek J. L. (2024). Perceptions and prevalence of anxiolytic medication usage for performance enhancement among musicians. Med. Probl. Perform. Art. 39, 155–161. doi: 10.21091/mppa.2024.04018, PMID: 39641564

[ref17] Liang C.-W. Huang Y.-S. Hung F.-C. (2021). Apprehension about the future: investigating the phenomenological characteristics of episodic future thinking in socially anxious adolescents. J. Behav. Ther. Exp. Psychiatry 73:101668. doi: 10.1016/j.jbtep.2021.101668, PMID: 34139637

[ref18] Liu H. (2023). Influence of dramatic performers with psychological anxiety on stage performance. CNS Spectr. 28, S103–S104. doi: 10.1017/S1092852923005126

[ref19] MacLeod A. K. Tata P. Kentish J. Carroll F. Hunter E. (1997). Anxiety, depression, and explanation-based pessimism for future positive and negative events. Clin. Psychol. Psychother. 4, 15–24.

[ref20] MacLeod A. K. Tata P. Kentish J. Jacobsen H. (1997). Retrospective and prospective cognitions in anxiety and depression. Cogn. Emot. 11, 467–479. doi: 10.1080/026999397379881

[ref21] Nicholson D. R. Cody M. W. Beck J. G. (2015). Anxiety in musicians: on and off stage. Psychol. Music 43, 438–449. doi: 10.1177/0305735614540018

[ref22] Niering M. Monsberger T. Seifert J. Muehlbauer T. (2023). Effects of psychological interventions on performance anxiety in performing artists and athletes: a systematic review with meta-analysis. Behav. Sci. 13:910. doi: 10.3390/bs13110910, PMID: 37998657 PMC10669558

[ref23] Nolen-Hoeksema S. Wisco B. E. Lyubomirsky S. (2008). Rethinking rumination. Perspect. Psychol. Sci. 3, 400–424. doi: 10.1111/j.1745-6924.2008.00088.x, PMID: 26158958

[ref24] Papageorgiou C. (2006). “Worry and rumination: styles of persistent negative thinking in anxiety and depression” in Worry and its psychological disorders: theory, assessment and treatment, eds. Davey G. C. L. Wells A. (Sussex, UK: Wiley). 21–40.

[ref25] Patston T. Loughlan T. (2014). Playing with performance: the use and abuse of beta-blockers in the performing arts. Vict. J. Music Educ. 1, 3–10. doi: 10.3316/ielapa.006954228695040

[ref26] Schacter D. L. Addis D. R. Hassabis D. Martin V. C. Spreng R. N. Szpunar K. K. (2012). The future of memory: remembering, imagining, and the brain. Neuron 76, 677–694. doi: 10.1016/j.neuron.2012.11.001, PMID: 23177955 PMC3815616

[ref27] Schacter D. L. Benoit R. G. Szpunar K. K. (2017). Episodic future thinking: mechanisms and functions. Curr. Opin. Behav. Sci. 17, 41–50. doi: 10.1016/j.cobeha.2017.06.002, PMID: 29130061 PMC5675579

[ref28] Wu J. Q. Szpunar K. K. Godovich S. A. Schacter D. L. Hofmann S. G. (2015). Episodic future thinking in generalized anxiety disorder. J. Anxiety Disord. 36, 1–8. doi: 10.1016/j.janxdis.2015.09.005, PMID: 26398003 PMC4658269

[ref29] Zakaria J. B. Musib H. B. Shariff S. M. (2013). Overcoming performance anxiety among music undergraduates. Procedia Soc. Behav. Sci. 90, 226–234. doi: 10.1016/j.sbspro.2013.07.086

